# Sleep Disturbance and Its Association With Purchasing Behavior of COVID-19 Medicine Among the Public After the Adjustment of Zero-COVID Policy in China: Results From a Web-Based Survey Study

**DOI:** 10.2196/79903

**Published:** 2026-01-06

**Authors:** Yu Huang, Xiang Zhao, Lei Wang, Qiaohong Lv, Qingqing Wu, Shuiyang Xu, Xuehai Zhang, Shuxian Wu

**Affiliations:** 1 Department of Health Education, Zhejiang Provincial Center for Disease Control and Prevention, Hangzhou, China; 2 Central Office, Zhejiang Provincial Center for Disease Control and Prevention, 630 Xincheng Road, Binjiang District, Hangzhou, Zhejiang, 310051, China, 86 057187115030

**Keywords:** adjustment of zero-COVID policy, China, COVID-19, drug purchasing, drug shortage, sleep disturbance

## Abstract

**Background:**

In December 2022, in light of the weakened pathogenicity of the new variants and other scientific considerations, China optimized its zero-COVID policy. As the situation evolved, the virus spread more widely across the country.

**Objective:**

This study aims to explore the public’s sleep status and its association with purchasing behavior of COVID-19 medicine after the adjustment of zero-COVID policy in China.

**Methods:**

A cross-sectional, internet-based survey among residents aged 18‐69 years was conducted in Zhejiang province, China, from December 16 to 30, 2022, to collect data on sociodemographic characteristics, COVID-19 drug purchasing behavior, sleep disturbance levels, etc. Chi-square tests, univariate analyses, and multivariate analyses were used to explore the associations among these factors.

**Results:**

Out of 38,480 participants, 20,803 (54.1%) reported sleep disruption after China’s COVID-19 response policy adjustment. The degree of impact varied, with 10,964 (52.70%) reporting “slight,” 3105 (14.93%) “moderate,” 3493 (16.79%) “significant,” and 3241 (15.58%) “very significant” impacts. Only 20.90% (782/3742) of those who deemed purchasing unnecessary had sleep disruptions, compared to 45.19% (6214/13,752) of those who acquired medications and 65.79% (13,807/20,986) of those who tried but failed to obtain them. Sleep disturbance levels were significantly associated with sociodemographic factors like age, education levels, occupation, marital status, and presence of family members diagnosed with COVID-19 (*P*<.05). By age, sleep disturbance proportions differed notably: 36.32% (409/1126) for those under 20 years, 54.81% (19,714/35,970) for the 20 to 60 age group, and 49.13% (680/1384) for individuals over 60 years. For education level, the proportions were 57.44% (517/900, primary school), 54.34% (3928 /7229, junior high school), 54.27% (3808/7017, senior high school), 53.99% (11,974/22,180, junior college/undergraduate), and 49.91% (576/1154, master’s degree), showing a clear downward trend as education level increased. By occupation, farmers had the highest rate (855/1447, 59.09%), followed by business/service industry workers and stay-at-home/unemployed individuals (13,925/24,750, 56.26%) and government staff (4161/7712, 53.95%), while 1242 out of 3049 (40.73%) health workers and 620 out of 1522 (40.74%) students had lower rates. Married participants had a 55.21% (17,143/31,053) sleep disturbance rate, and those with COVID-positive family members had the highest rate (2023/2873, 70.41%). Multivariate logistic regression, adjusting for these sociodemographic factors, showed that compared to those who thought purchasing COVID-19 medications was unnecessary, those who acquired medications were 3.11 times (adjusted odds ratio 3.11, 95% CI 2.85‐3.39) more likely, and those who tried but couldn’t get medications were 7.11 times (adjusted odds ratio 7.11, 95% CI 6.53‐7.74) more likely to experience sleep disturbance.

**Conclusions:**

The adjustment of China’s zero-COVID policy affected the sleep health of the public, which was closely linked to drug-purchasing status, especially among the older people, those with lower education levels, and those with family members diagnosed with COVID-19. It highlights the need to develop and deploy interventions aimed at promoting better sleep health in times of crisis.

## Introduction

In December 2019, an unprecedented pneumonia outbreak of unknown origin emerged in Wuhan, Hubei province, China [1,2]. Subsequently identified as caused by the novel coronavirus (COVID-19), this highly contagious pathogen rapidly spread beyond China’s borders within just 30 days [3-6]. Characterized by potentially severe symptoms such as acute respiratory failure and sepsis [7], COVID-19 posed a significant threat to global public health, prompting the World Health Organization (WHO) to declare it a public health emergency of international concern on January 30, 2020 [8].

To contain the virus, China implemented the dynamic zero-COVID policy, which included strict measures like national lockdowns, school closures, travel restrictions, and the suspension of numerous economic activities [9-13]. However, as the virulence of new virus variants decreased and COVID-19 vaccines became more prevalent, China adjusted its policy on December 7, 2022. The mandatory large-scale nucleic acid testing requirements, the centralized isolation for asymptomatic cases and mild symptomatic patients, and the mobility restrictions were removed [14,15]. This suddenly loosened policy led to a change in the epidemic situation, with a wider spread of the virus across the country.

The COVID-19 pandemic has had a profound impact on people’s lives, particularly on their mental health and sleep quality [16-20]. A large-scale global study (combining 250 studies with nearly 500,000 participants from 49 countries) found that about 40% of people had sleep disturbances during the COVID-19 pandemic (95% CI 37.56%-43.48%). Among specific populations, the prevalence of sleep problems varied, with 52.39% (95% CI 41.69%-62.88%) among COVID-19 patients, 45.96% (95% CI 36.90%-55.30%) among children and adolescents, 42.47% (95% CI 37.95%-47.12%) among health care workers, 41.50% (95% CI 32.98%-50.56%) among special populations with health care needs, 41.16% (28.76%-54.79%) among university students, and 36.73% (32.32%-41.38%) among the general population [21]. Research in China shows that the prevalence of insomnia during the early and late stages of the pandemic was 37.0% (95% CI 34.1%‐39.9%) and 41.8% (95% CI 33.6%‐50.0%) respectively. Notably, health care workers, COVID-19 patients, those with chronic diseases, and people with mental disorders are more likely to experience insomnia than the general population [22].

Sleep is a fundamental physiological process that is essential for maintaining physical and mental health [19,23]. It plays a crucial role in various bodily functions, including homeostasis, immune system regulation, cognitive function, neural plasticity, memory consolidation, energy maintenance, macromolecular biosynthesis, and metabolism regulation [24,25]. Conversely, sleep deprivation can lead to a range of adverse health outcomes, such as impaired physical and mental performance, increased risk of depression, stroke, chronic inflammation, cancers, and weakened immune defense, which may be particularly relevant during the COVID-19 pandemic [26,27].

During the pandemic, multiple factors influenced people’s sleep quality. The spread of news and rumors through social media, television, and daily interactions about COVID-19’s severity and fatality numbers fueled anxiety and fear. Additionally, lack of in-person social connections, changes in eating patterns, less outdoor activities which lead to limited exposure to sunlight, and consequently extended exposure to blue and bright light from screens may lead to disturbed circadian rhythms [21,28-32]. Above all, the suddenly loosened policy and the increased risk of infection may lead some individuals to panic-purchase medical resources—especially COVID-19 medicines (including cough medicine, nasal congestion/snot relievers, fever reducers, pain relievers, and other symptom-alleviating pharmaceuticals for COVID-19)—to ensure timely administration when needed [33,34]. The purchasing behavior towards COVID-19 medicines reflected people’s perception of the disease and their self-protection strategies. However, it may also leave those in need unable to obtain them. This behavior was not only driven by the actual need for medications but also by psychological factors, such as the desire for a sense of security in the face of an uncertain health situation. Understanding the relationship between sleep status and these factors after the policy adjustment is of great significance for public health.

However, there is a lack of in-depth research on how the adjustment of China’s zero-COVID policy affects the public’s sleep status and its connection with the purchasing behavior of COVID-19 medicine. This study aims to fill this gap by conducting a cross-sectional, internet-based survey to assess the public’s sleep status and explore its association with the purchasing behavior of COVID-19 medicine after the policy adjustment. The findings of this research can provide valuable insights for public health interventions and future pandemic-related policies.

## Methods

### Questionnaire

The questionnaire was formulated by the Zhejiang Provincial Center for Disease Control and Prevention based on pandemic-specific context, literature review, and expert consultation. To ensure its reliability, a pilot study was first conducted. Following the feedback from the pilot study and through two rounds of collaboration based on the Delphi method, the questionnaire was revised. During this process, subject matter experts evaluated its face validity, and a literature review was carried out to assess its content validity.

The questionnaire collected sociodemographic data: purchasing behavior of COVID-19 medications; specific types of COVID-19 drugs that respondents had purchased; channels through which they bought these medications; and degrees of their sleep disturbances. Sociodemographic data includes age, gender, education level, occupation, marital status, and diagnosis of COVID-19 among family members. The specific questions regarding the purchasing behavior of COVID-19 medications and the degrees of their sleep disturbances were as follows: (1) “Have you purchased COVID-19 drugs?” (2) “Which of the following COVID-19 drugs have you purchased?” (3) “Through what channel did you successfully purchase these medicines?” (4) “In the past week, has the adjustment of COVID-19 policies affected your sleep?” No item randomization or adaptive questioning was used, and the 18-item questionnaire was distributed across 3 screens. Participants could review and revise answers via a “Back” button on each screen before submission. IP address tracking was used to prevent multiple entries from the same individual, and no two entries from the same IP address were allowed within 72 hours.

To maintain the quality of the survey data, a quality control question was incorporated into the questionnaire to identify survey respondents who did not answer the questions seriously. The question was, “This is a quality control question, please choose B.” All the survey subjects who chose other options were not included in the final data analysis. In addition, server-side completeness checks were implemented upon submission, incomplete responses were rejected, and participants were prompted to fill in missing fields.

The questionnaire was developed and administered entirely in simplified Chinese, consistent with the native language of the target population—see Supplemental File for Chinese (in Multimedia Appendix 1) and translated English version (in Multimedia Appendix 2) of the questionnaire.

### Recruitment

The survey was carried out between December 16 and 30, 2022, among residents aged 18-69 years in Zhejiang province, China. Initially, an online open survey questionnaire was dispatched to the 11 municipal Centers for Disease Control and Prevention. These centers then actively promoted and conducted the online investigation by sharing survey links via local official WeChat public accounts, neighborhood WeChat groups, and other regional lifestyle apps. Visitors to the survey link could choose to exit at any time, and no access to other content was restricted to survey completion. The questionnaire was hosted on Wenjuan, a professional web-based platform specialized in questionnaires, tests, assessments, and voting. This platform is dedicated to offering users powerful and user-friendly services such as online questionnaire design, data collection, customized reports, and analysis of survey results.

### Measurements Control

#### Levels of Sleep Disturbance

The Likert scale was used to assess the levels of sleep disturbance related to the COVID-19 epidemic reported by participants. In our research, we used a 5-point Likert scale which consists of 5 answer options: (1) not at all, (2) slight, (3) moderate, (4) significant, and (5) very significant.

#### Purchasing Behavior of COVID-19 Medicine

Purchasing behavior of COVID-19 medicine was classified as follows: (1) participants who felt it unnecessary to purchase COVID-19 medicine, (2) participants who had tried, but were unable to access any COVID-19 medicine, and (3) participants who had successfully purchased COVID-19 medicine.

Participants were classified as having COVID-19 medication purchasing behavior during the early phase following China’s zero-COVID policy adjustment if they had acquired the following drugs at that time: (1) cough medicine, (2) nasal congestion/snot relievers, (3) fever reducers, (4) pain relievers such as ibuprofen (Advil, Motrin IB, and others) or acetaminophen (Tylenol and others), and (5) other pharmaceuticals capable of alleviating COVID-19 infection symptoms.

#### Family Members Infected With COVID-19

“Family members infected with COVID-19” was defined as "individuals who reside in the same household as the participant and had been diagnosed with COVID-19 (via nucleic acid or antigen testing) at any time prior to the survey.”

### Statistical Analysis

Data were exported from the Wenjuan website to Excel (Microsoft), and were analyzed using the Statistical Analysis System (SAS), version SAS Viya Long-Term Support 2024.03 (SAS Institute Inc., Cary, NC, USA). Standard descriptive statistics were used for continuous and categorical variables to describe the characteristics of participants in this study. The *χ*² test was used to explore the association between the purchasing behavior of COVID-19 medicines and the levels of sleep disturbance. Univariate logistic regression analysis was conducted to identify the sociodemographic factors associated with sleep disturbance. We used multivariate logistic regression to identify the association between sleep disturbance and purchasing behavior of COVID-19 medicines (categorical variable; reference group was “deemed unnecessary to purchase;” comparison groups was “successfully purchased” and “tried but failed to purchase”). The initial candidate variables included in the model were: age, gender, education level, occupation, marital status, and diagnosis of COVID-19 among family members. A backward stepwise selection approach was used, with a significance level of *P*<.10 for variable retention. Odds ratios (OR) with 95% CI were used to express measures of the associations. *P* values less than .05 were considered to represent significance (two-sided).

### Ethical Considerations

The Zhejiang Provincial Center for Disease Control and Prevention Ethics Board approved the study protocol (2022-027-01). Conducted under strict anonymity, the survey safeguarded the privacy of all participants. No personally identifiable information was collected at any stage of the survey, the dataset was stored by specified staff, and all researchers involved signed a data confidentiality agreement. Informed consent was obtained from all participants prior to their inclusion in the study. By voluntarily clicking an electronic consent box, participants provided their informed consent, thereby indicating their willingness to participate in this research project. As compensation, participants could collect free health education materials at their local Center for Disease Control and Prevention.

## Results

### Respondents’ Characteristics

A total of 40,130 residents took part in the survey, and after implementing quality control procedures, 1650 samples were excluded, with 38,480 samples ultimately included in the study. The sample consisted of 10,431 (27.11%) male participants and 28,049 (72.89%) female participants, with 1129 (2.93%) participants aged 20 years or below, 35,970 (93.48%) participants aged between 20 and 60, and 1384 (3.60%) participants over 60. Regarding occupations, 3049 (7.92%) were health workers, 7712 (20.04%) were government staff, 1522 (3.96%) were students , 1447 (3.76%) were farmers, and 24,750 (64.32%) were business/service industry workers and stay-at-home/unemployed individuals. In terms of education level, 22,180 (57.64%) participants had an education of junior college or undergraduate. Marital status showed that 31,053 (80.70%) people were married and 7427 (19.30%) people were unmarried, while 2873 (7.47%) people resided in households with confirmed COVID-19 cases. The detailed demographic characteristics of the participants are presented in Table 1.

**Table 1.** Sociodemographic characteristics of participants completing the online survey in the early stage after the lifting of zero-COVID policy in China (n=38,480).

Characteristic	Count, n (%)
Age (y)^a^	
<20	1126 (2.93)
20‐60	35,970 (93.48)
>60	1384 (3.60)
Gender	
Male	10,431 (27.11)
Female	28,049 (72.89)
Occupation	
Health workers	3049 (7.92)
Government staff	7712 (20.04)
Students	1522 (3.96)
Farmers	1447 (3.76)
Business/service industry workers and stay-at-home/unemployed individuals	24,750 (64.32)
Education level	
Primary school	900 (2.34)
Junior high school	7229 (18.79)
Senior high school	7017 (18.24)
Junior college/undergraduate	22,180 (57.64)
Postgraduate	1154 (3)
Family members infected with COVID-19	
Yes	2873 (7.47)
No	35,607 (92.53)
Marital status	
Married	31,053 (80.70)
Others (single/divorced/widowed/separated, etc)	7427 (19.30)

a In the preliminary analysis, participants were divided into 10-year age groups (<20, 20‐29, 30‐39, 40‐49, 50‐59, 60‐69 y). Given similar purchasing behavior in 20‐29 to 50‐59 years groups, these 4 groups were merged into 20‐60 years for concise result presentation, with this data-driven rationale added herein.

### The Impact of China’s Zero-COVID Policy Adjustment on the Sleep Status of the Public of Zhejiang, China

When the Likert scale was used to assess the degree of impact on participants’ sleep, the findings revealed that out of 38,480 participants, 17,677 (45.94%) indicated that the adjustment of China’s COVID-19 response policies had no effect on their sleep. In contrast, 20,803 (54.06%) reported experiencing varying levels of sleep disruption. Specifically, 10,964 (52.70%) described the impact as “slight,” 3105 (14.93%) as “moderate,” 3493 (16.79%) as “significant,” and 3241 (15.58%) as “very significant.”

### Association Between Sleep Status and Purchasing Behavior of COVID-19 Medicine Among the Public in the Early Stage After the Adjustment of China’s Zero-COVID Policy

As shown in Table 2, among individuals who had tried, but were unable to access any COVID-19 medicine, 34.21% (7179/20,986) reported no impact on their sleep by the lifting of China’s zero-COVID policy in the past week. In contrast, 54.81% (7538/13,752) of those who successfully acquired COVID-19 medications indicated no sleep disturbances. Notably, the proportion of individuals who deemed it unnecessary to buy COVID-19 medications and experienced no sleep disruption was as high as 79.10% (2960/3742).

**Table 2.** Impact of purchasing behavior of the COVID-19 medicine on the sleeping status among the participants of an online survey in the early stage after the lifting of zero-COVID policy in China.

Purchasing of the COVID-19 medicine	How has your sleep been affected by the lifting of China’s zero-COVID policy in the past week^a^? n (%)					Total
	Very significant	Significant	Moderate	Slight	Not at all	
Participants who felt it unnecessary to purchase COVID-19 medicine	60 (1.60)	62 (1.66)	99 (2.65)	561 (14.99)	2960 (79.10)	3742
Participants who purchased COVID-19 medicine	629 (4.57)	931 (6.77)	917 (6.67)	3737 (27.17)	7538 (54.81)	13,752
Participants who had tried, but were unable to access any COVID-19 medicine	2552 (12.16)	2500 (11.91)	2089 (9.95)	6666 (31.76)	7179 (34.21)	20,986

a *χ*=3639.2; *P*<.001.

Among the 3742 respondents who considered purchasing COVID-19 medications unnecessary, the numbers of those experiencing slight, moderate, significant, and very significant impacts on sleep were 561 (14.99%), 99 (2.65%), 62 (1.66%), and 60 (1.60%), respectively. Among the 13,752 participants who had successfully obtained COVID-19 medications, the corresponding figures were 3737 (27.17%), 917 (6.67%), 931 (6.77%), and 629 (4.57%). For the 20,986 individuals who attempted to purchase but failed to obtain COVID-19 medications, the numbers were 6666 (31.76%), 2089 (9.95%), 2500 (11.91%), and 2552 (12.16%). A *χ*² test for comparing multiple sample rates revealed that, overall, there were significant differences among the population rates (*P*<.001).

### Factors Associated With Sleep Disturbance Among the Public in the Early Stage After the Adjustment of China’s Zero-COVID Policy

As shown in Table 3, there were statistically significant differences in sleep disturbance after the adjustment of China’s zero-COVID policy among participants by age, education level, occupation, marital status, and diagnosis of COVID-19 among family members .

**Table 3.** Sociodemographic factors associated with sleep disturbance among the public in the early stage after the lifting of China’s zero-COVID policy.

Variables	Sleep status affected by the lifting of China’s zero-COVID policy, n (%)		OR^a^ (95%CI)
	Yes	No	
Sex			
Male	5716 (54.80)	4715 (45.20)	1.04 (0.99-1.09)
Female	15,087 (53.79)	12,962 (46.21)	Reference
Age (years)			
<20	409 (36.32)	717 (63.68)	Reference
20‐60	19,714 (54.81)	16,256 (45.19)	2.13 (1.88-2.41)^b^
>60	680 (49.13)	704 (50.87)	1.69 (1.44-1.99)^b^
Education level			
Primary school	517 (57.44)	383 (42.56)	1.36 (1.14-1.61)^b^
Junior high school	3928 (54.34)	3301 (45.66)	1.19 (1.05-1.35)^c^
Senior high school	3808 (54.27)	3209 (45.73)	1.18 (1.04-1.35)^c^
Junior college/undergraduate	11,974 (53.99)	10,206 (46.01)	1.17 (1.03-1.31)^c^
Postgraduate	576 (49.91)	578 (50.09)	Reference
Occupation			
Health workers	1242 (40.73)	1807 (59.27)	Reference
Government staff	4161 (53.095)	3551 (46.05)	1.71 (1.57-1.86)^b^
Students	620 (40.74)	902 (59.26)	1.00 (0.88-1.13)
Business or service industry/stay-at-home/unemployed, etc	13,925 (56.26)	10,825 (43.74)	1.87 (1.73-2.02)^b^
Farmers	855 (59.09)	592 (40.91)	2.10 (1.85-2.39)^b^
Marital status			
Married	17,143 (55.21)	13,910 (44.79)	1.27 (1.21-1.33)^b^
Others (divorced/widowed/separated, etc)	3660 (49.28)	3767 (50.72)	Reference
Has anyone in your family been diagnosed with COVID-19?			
Yes	2023 (70.41)	850 (29.59)	2.13 (1.96-2.32)^b^
No	18,780 (52.74)	16,827 (47.26)	Reference

a OR: odds ratio.

b *P*<.001.

c *P*<.01.

Age emerged as a notable predictor: participants aged 20‐60 years had the highest sleep disturbance rate (19,714/35,970, 54.80%), followed by those over 60 years (680/1384, 49.13%), while 409 out of 1126 participants under 20 years reported sleep disturbance (409/1126, 36.32%). Education level showed a clear inverse relationship with sleep disturbance: the rate was highest among primary school graduates (517/900, 57.44%) and gradually decreased with higher education, reaching 49.91% (576/1154) for master’s degree holders. Occupation also played a role. Farmers had the highest rate (855/1447, 59.09%), followed by business/service industry workers and stay-at-home/unemployed individuals (13,925/24,750, 56.26%), government staff (4161/7712, 53.95%), while 1242 out of 3049 health workers (1242/3049, 40.74%) and 620 out of 1522 students (620/1522, 40.74%) had lower rates. Marital status and family COVID-19 diagnosis further contributed to sleep disturbance: married participants had a higher (17,143/31,053, 55.21%) sleep disturbance rate than unmarried/separated individuals (OR 1.27, 95% CI 1.21‐1.33). Most notably, participants with family members diagnosed with COVID-19 had the highest sleep disturbance rate (2023/2873, 70.41%) across all groups, with odds of disturbance 2.13 times higher than those without infected family members (OR 2.13, 95% CI 1.96‐2.32) (detailed values in Table 3).

**Figure 1.**
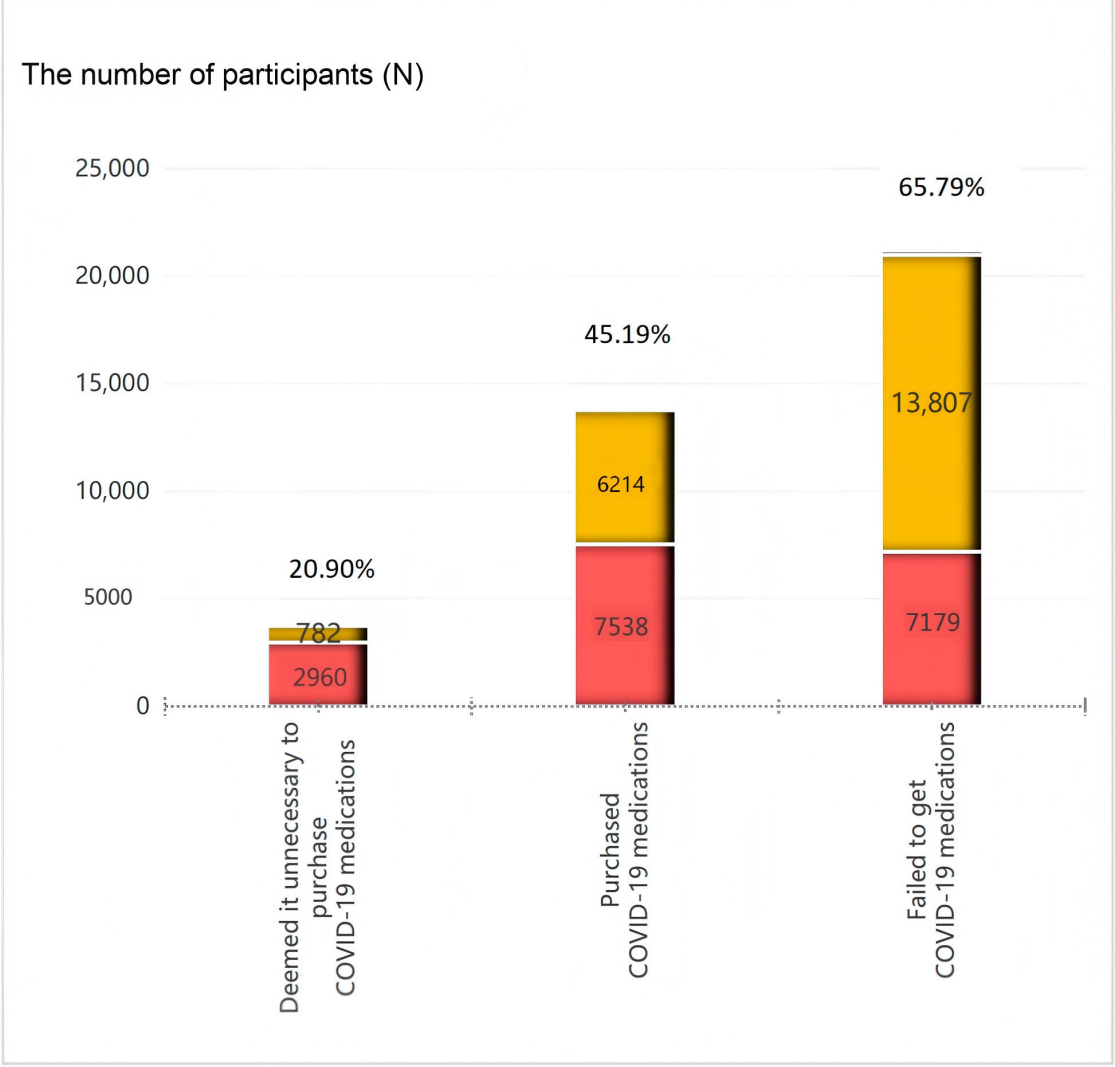
Stacked bar chart showing individuals affected by sleep disturbance stratified by COVID-19 medication access status among the public in the early stage after the lifting of China’s zero-COVID policy. The yellow color represents those who reported experiencing sleep disturbance, and the red color represents those unaffected.

As shown in Figure 1, among individuals who had tried, but were unable to access any COVID-19 medicine, 65.79% (13,807/20,986) reported experiencing sleep disturbance. In contrast, 45.19% (6214/13,752) of those who successfully acquired COVID-19 medications indicated sleep disturbances. Notably, the proportion of individuals who deemed it unnecessary to buy COVID-19 medications and experienced sleep disruption was a mere 20.9% (782/3742).

By using multivariate logistic regression analysis and after adjusting for variables including gender, age, education level, occupation, marital status, and presence of family members diagnosed with COVID-19, it was discovered that there was a statistically significant correlation between purchasing behavior of COVID-19 medicine and the sleep status of the participants. Table 4 shows that, compared to those who deemed it unnecessary to buy COVID-19 medications, those who successfully acquired COVID-19 medications were 3.11 times more likely to encounter sleep disturbance (adjusted odds ratio [aOR] 3.11, 95% CI 2.85‐3.39). Moreover, those who had tried, but were unable to access any COVID-19 medicine were 7.11 times (aOR 7.11, 95% CI 6.53‐7.74) more likely to experience sleep disturbance.

**Table 4.** Multivariate logistic analysis on the association between the sleep status and the purchasing of COVID-19 medicines among the public in the early stage after the lifting of zero-COVID policy in China.

Purchasing of COVID-19 medicine	Participants, n	Sleep status affected by COVID-19 epidemic, n (%)	aOR^a^ (95% CI)	*P* value
Participants who felt it unnecessary to purchase COVID-19 medicine	3742	782 (20.90)	Reference	—^b^
Participants who purchased COVID-19 medicine	13,752	6214 (45.19)	3.11 (2.85-3.39)	<.001
Participants who had tried, but were unable to access any COVID-19 medicine	20,986	13,807 (65.79)	7.11 (6.53-7.74)	<.001

a aOR: adjusted odds ratio.

b Not applicable.

## Discussion

### Principal Findings

This study explores the sleep status of the public in Zhejiang province, China, following the adjustment of the zero-COVID policy and explores its relationship with the purchasing behavior towards COVID-19 medicine. The results offer valuable insights into the post-policy-adjustment situation. Given the dearth of similar studies, this research will serve as a foundation for future studies that highlight such crucial issues in the context of future public health emergencies.

After the zero-COVID policy adjustment, news and rumors about infection severity and fatalities—spread via social media, television, and daily interactions—fueled public anxiety and fear. Consequently, people act on their primal instincts and are prone to engage in panic buying to ease this psychological reaction [35]. The results of our study revealed that 35.74% (13,752/38,480) of the participants had successfully purchased COVID-19 medicines, 54.54% (20,986/38,480) had attempted to make a purchase but were unable to obtain any of the drugs, and 9.72% (3742/38,480) felt it was unnecessary to stockpile COVID-19 drugs. Psychologically, those who tried to purchase COVID-19 medicines may be driven by a fear of drug shortages, price increases, the surge in infections, and the availability of medicines and home delivery services during the pandemic [34,36]. Therefore, whether they successfully purchase medications directly affects their psychological state. Those who manage to buy medications will experience a certain degree of anxiety relief, while those who fail to do so may become more anxious [37-40].

Our study revealed that 54.06% (20,803/38,480) of the participants experienced sleep disturbances following the adjustment of the zero-COVID policy. It can be explained as a psychological burden during stressful circumstances. Pandemic-related sleep disturbance was also confirmed by many other studies worldwide. A study carried out in Beijing, China, found that, during the second wave of COVID-19, the overall prevalence of sleep disturbance was 50.8% [41]. Madeleine reported a 33.5% sleep disturbances during the COVID-19 shutdown phase in Germany [42]. It is also documented that there is a negative impact on the quality of sleep among the Spanish population during the lockdown period due to COVID-19 [43]. Studies found that COVID-19 had both negative and positive impacts on adolescent sleep. A study in Canada found that the shutdown of schools due to the COVID-19 pandemic might lead to a 2-hour shift in the sleep of typically developing adolescents, longer sleep duration, improved sleep quality, and less daytime sleepiness compared to those experienced under the regular school-time schedule [44]. Parents of 8th grade students from local schools across Ohio, Kentucky, and Virginia (USA) reported adolescents had more difficulties initiating and maintaining sleep during COVID-19 than before COVID-19, with clinically elevated rates increasing from 24% to 36%. Both bedtimes and wake times shifted later during COVID-19, and adolescents reported more delayed sleep/wake behaviors. Adolescents also reported less daytime sleepiness and longer school night sleep duration during COVID-19 [45].

Our study found that the degree of sleep disturbance varied, with 52.70% (10,964/20,803) reporting “slight,” 14.93% (3105/20,803) “moderate,” 16.79% (3493/20,803) “significant,” and 15.58% (3241/20,803) “very significant.” It suggests a complex and diverse range of effects on individuals, which may be related to various factors. Our study found that sociodemographic factors played a crucial role in sleep disturbances. Older people were more likely to experience sleep disturbances compared to the younger. A systematic review found that studies involving older participants had a higher pooled prevalence of insomnia symptoms during the COVID-19 pandemic [22]. Older individuals may be more sensitive to policy change impacts, due to preexisting health conditions, limited medical access, and greater reliance on stable daily routines—all of which increase their fear of infection [46]. Even though women were found to be more prone to sleep disturbance than men [47-49], the perceived impact of COVID-19 on sleep was not different between sexes in our study, which was also confirmed by research from the United States by Jeremy A Bigalke [50]. Literature reviews also revealed that sex had no bearing on the estimated prevalence of sleep disturbance [21]. Education level was inversely related to sleep disturbances; as the education level increased, the likelihood of experiencing sleep disturbances decreased. This could be because individuals with higher education levels are generally better at accessing and understanding information and medical resources, and thus better able to cope with the stress caused by the pandemic. Studies found that people with higher socioeconomic status are more likely to attain medical resources during the COVID-19 pandemic [40,51]. While health care workers were documented as the most sleep-vulnerable group early in the COVID-19 pandemic [52,53], our study found the opposite in post-policy-adjustment in China. Different occupations face varying degrees of stress during the pandemic. For example, government staff may be burdened with epidemic-prevention work responsibilities, while business/service industry workers may worry about economic losses, and farmers may be concerned about the impact of the pandemic on agricultural production. However, at the very beginning after the adjustment of zero-COVID policy in China, the anxiety of getting infected without drugs in hand might relate to poor sleep quality. Our previous research found that 56.54% of health workers had successfully purchased COVID-19 medicines, which was the highest among all the occupation groups and might explain the less experience of sleep disturbance in this study [40]. Marital status and family members’ COVID-19 diagnosis also had an impact. Married individuals had a higher probability of sleep disturbances, perhaps due to increased family responsibilities during the pandemic. Participants with family members diagnosed with COVID-19 were more prone to sleep disturbances, with a proportion as high as 70.41% (2023/2873), indicating that the direct impact of the disease on the family can cause significant psychological pressure, which was also confirmed by a study carried out in Bangladesh. Participants having relatives or acquaintances infected with COVID-19 were more likely to experience poor sleep quality (68.8% vs 49.0%) [47].

In addition to sociodemographic factors, our study found a strong association between the purchasing behavior of COVID-19 medicine and sleep status. Those who deemed purchasing unnecessary had the lowest proportion of sleep disruptions, while those who tried but failed to obtain medications had the highest. After adjusting for sociodemographic factors, it demonstrated that compared to those who thought purchasing was unnecessary, those who acquired medications were 3.11 times more likely, and those who tried but couldn’t get medications were 7.11 times more likely to experience sleep disturbance. This difference highlights the role of medication availability and the psychological stress related to it [35]. For those who manage to buy the medicine, it may bring a sense of security and relieve some anxiety, as they feel prepared for potential infections. However, their higher sleep disturbance rate—compared to those who saw no need to purchase COVID-19 medicines—suggests lingering concerns about the disease and future drug shortages still impact their mental state. For those who fail to purchase it, the situation can exacerbate their anxiety. The fear of not being able to access necessary medications during illness and the uncertainty about their health can heighten stress levels, making them more likely to experience sleep disturbance. Our previous research revealed an inverse correlation between anxiety levels and the behavior of purchasing COVID-19 medications which can further explain the association between purchasing behavior of COVID-19 medicine and sleep status. Compared to those with severe anxiety, those with moderate anxiety were 1.76 times more likely to have purchased COVID-19 medicine (aOR 1.76, 95% CI 1.64‐1.89); those with mild anxiety were 2.11 times (aOR 2.11, 95% CI 1.98‐2.24) more likely to have purchased COVID-19 medicine; those with no anxiety were 2.48 times (aOR 2.48, 95% CI 2.31‐2.67) more likely to have purchased COVID-19 medicine [40]. Individuals who were unable to obtain COVID-19 medications may have experienced heightened anxiety, which in turn could have contributed to increased instances of sleep disturbance. The very strong aOR of 7.11 for those unable to access medications and their likelihood of reporting sleep disturbances is notable. However, this causal inference requires further validation to account for unmeasured confounders (eg, baseline anxiety levels, preexisting sleep disorders).

### Limitations

This study has certain limitations. Firstly, to quickly explore the association between sleep disturbance and COVID-19 medicine purchasing behavior, the questionnaire was kept concise, omitting potential confounders (eg, COVID-19 vaccination status, comorbidities). “Participants who had tried, but were unable to access any COVID-19 medicine” was not clearly defined, which may lead to misclassification of participants’ purchasing behavior. Sleep disturbance was measured via a single Likert-scale item—practical for large surveys but lacking details on sleep frequency/duration/severity, limiting study depth. These should be explored in future studies. Secondly, the data collection relied on self-reported information, which is subject to recall bias and may distort the study results. Thirdly, among the participants in the online survey, the participation of women and those with a higher education level is significantly higher than that of men and those with a low education level. Thus, applying the survey results directly to the general population could lead to inaccuracies.

### Conclusions

The findings reflect 54.1% of participants experiencing varying levels of sleep disturbance after the zero-COVID policy adjustment in Zhejiang, China. The older people, those with lower education levels, those with family members diagnosed with COVID-19, and those who had tried, but were unable to access any COVID-19 medicine were mostly affected. Public health agencies should design tailored communication campaigns for different demographic groups, especially for those high-risk groups, to minimize COVID-19-related fear and anxiety. Additionally, optimizing the supply and distribution of COVID-19 medications might help alleviate the stress of individuals who are unable to access them. A robust, transparent pharmaceutical supply chain monitoring system and dedicated channels (hotlines/online platforms) for reporting shortages would enable timely supply adjustments, ensuring high-risk groups access necessary drugs. Beyond China, our findings hold global relevance to observations in other countries where postpandemic policy shifts linked to anxiety and sleep disruptions [42,43].

## Supplementary material

10.2196/79903 Multimedia Appendix 1 Questionnaire: Chinese version.

10.2196/79903 Multimedia Appendix 2 Questionnaire: translated English version.

## Abbreviations

aOR: adjusted odds ratio
OR: odds ratio
SAS: Statistical Analysis System
