# Supplementary material for: Sleep Disturbance and Its Association With Purchasing Behavior of COVID-19 Medicine Among the Public After the Adjustment of Zero-COVID Policy in China: Results From a Web-Based Survey Study
Source: Interact J Med Res. 2026 Jan 6;15:e79903. doi: 10.2196/79903 (PMC12774404; doi:10.2196/79903)
Supplement: Multimedia Appendix 1 [file ijmr-v15-e79903-s001.doc]

**新冠疫情下浙江省居民家庭药物购买情况调查**

尊敬的居民朋友：

您好！近期，我国对新冠防控政策进行了调整，生产生活秩序正逐步恢复。新冠病毒变异株的致病力虽有所减弱，但传染性更强。为了解居民家庭新冠相关药物的购买情况，以及大家针对政策调整的心理与行为反应，从而更好地优化全省药品调配工作，我们诚邀您参与本次调查。您的个人信息及回答将严格保密。为表感谢，您可前往当地疾病预防控制中心领取免费健康教育资料。若您同意参与，请点击下方电子同意框，随后开始填写问卷。

同意参与 🞎

不同意参与 🞎

**1.性别：**

A. 男

B. 女

**2.年龄：**

A.20 岁及以下

B.21-30 岁

C.31-40 岁

D.41-50 岁

E.51-60 岁

F.60 岁及以上

**3.文化程度：**

A.小学及以下

B.初中

C.高中 / 职高 / 中专

D.大专及本科

E.硕士及以上

**4.职业：**

A.政府 / 事业单位工作人员

B.企业 / 商业 / 服务业工作人员

C.农民（含农林牧副渔业及农民工）

D.离退休人员

E.家庭主妇 / 主夫

F.学生

G.无业

F.医务人员

H.其他

**5.婚姻状况：**

A.未婚

B.已婚

C.离异

D.其他

**6.与您同住的家庭成员人数：______人**

**7.您家中是否有老人或小孩？**

A.有老人，无小孩

B.有小孩，无老人

C.既有老人也有小孩

D.均无

**8.调查前，您的同住家庭成员中是否有人经核酸或抗原检测确诊过新冠病毒感染？**

A.是

B.否

1. **您是否购买过新冠相关药物？**
2. 是
3. 否（跳转至第 12 题）

**10.您购买的新冠相关药物包括以下哪些？（可多选）**

A. 缓解发热、疼痛症状的药物，如布洛芬、连花清瘟、阿司匹林、含对乙酰氨基酚类药物（如散利痛、扑热息痛等）

B. 缓解流涕、鼻塞、打喷嚏等感冒症状的药物，如泰诺、白加黑、维 C 银翘片等

C. 缓解咳嗽、咳痰症状的药物，如川贝枇杷膏、枇杷露、急支糖浆、甘草口服液、肺力咳等

D. 缓解恶心、呕吐、腹泻等消化道症状的药物，如藿香正气液 / 胶囊、止泻药

E. 其他药物

**11.您通过哪些渠道成功购买了这些药物？（可多选）**

A. 医院

B. 药店

C. 网络平台

D. 其他渠道

**12.您未购买新冠相关药物的原因是什么？**

A. 尝试过购买，但未能买到

B. 认为无需购买

**13.过去一周，新冠政策调整是否影响了您的睡眠？**

A. 是

B. 否（跳转至第 16 题）

**14.您认为新冠政策调整对您睡眠状况的影响程度如何？**

A. 影响极大

B. 影响较大

C. 影响一般

D. 影响较小

**15.您认为新冠政策调整背景下，导致您睡眠受影响的具体原因有哪些？（可多选）**

A. 担心新冠应对物资（如药品）不足

B. 担心自身感染

C. 担心家庭成员感染

D. 担心疫情影响工作、生意、学习等

E. 其他：______

**16.未来两个月，您认为自己或家庭成员感染新冠病毒的可能性有多大？**

A. 肯定会感染

B. 大概率会感染

C. 可能会感染

D. 不太可能感染

E. 肯定不会感染

**17.若您居家经核酸或抗原检测确诊新冠病毒感染，您会怎么做？**

A. 立即前往医院

B. 居家观察，出现症状后再去医院

C. 居家观察，仅在症状严重且自行用药无法缓解时前往医院

**18.过去两周，您是否有过以下行为？（可多选）**

A. 频繁查看新冠相关信息

B. 频繁洗手

C. 更严格地佩戴口罩

D. 想方设法多购买相关物品（如口罩、抗原检测试剂盒、药品等）

E. 改变出行方式，减少乘坐公共交通工具

F. 接种新冠疫苗

G. 尽量居家不外出

H. 反复进行检查（如核酸检测、体温测量、就医等）

I. 无上述情况
