# Supplementary material for: Sleep Disturbance and Its Association With Purchasing Behavior of COVID-19 Medicine Among the Public After the Adjustment of Zero-COVID Policy in China: Results From a Web-Based Survey Study
Source: Interact J Med Res. 2026 Jan 6;15:e79903. doi: 10.2196/79903 (PMC12774404; doi:10.2196/79903)
Supplement: Multimedia Appendix 2 [file ijmr-v15-e79903-s002.doc]

**Survey on Household Covid-19 Medicine Purchasing Behaviour**

Dear Residents,

How are you! Recently, China has adjusted its Zero-COVID policy, gradually resuming production and daily life. Although the pathogenicity of the COVID-19 variant has weakened, its transmissibility has become stronger. To understand the current status of household covid-19 medicine purchasing status and residents' psychology and behaviour responses toward the adjustment, so as to better optimize drug allocation across the province, we invite you to participate in this survey. Your personal information and responses will be strictly confidential. We appreciate your support for our work! As compensation, you could collect free health education materials at their local Center for Disease Control and Prevention. If you agree to participate, please click the following electronic consent box, and start with the survey.

Agree to participate 🞎

Disagree to participate 🞎

1.Gender:

1. Male
2. Female

2.Age:

1. 20 years old and below
2. 21-30 years old
3. 31-40 years old
4. 41-50 years old
5. 51-60 years old
6. 60 years old and above
7. Educational background:
8. Primary school or below
9. Junior high school
10. High school/vocational high school/technical secondary school
11. College/university undergraduate
12. Master's degree or above
13. Occupation:
14. Government/public institution staff
15. Enterprise/commercial/service industry staff
16. Farmer (including agriculture, forestry, animal husbandry, fishery, and migrant workers)
17. Retired
18. Housewife/husband
19. Student
20. Unemployed
21. Medical staff
22. Other

5.Marital status:

1. Single
2. Married
3. Divorced
4. Others

6.Number of family members who reside in the same household as you: ______

1. Are there any elderly people or children in your family?
2. Elderly people, no children
3. Children, no elderly people
4. Both elderly people and children

D. Neither

1. Has anyone who reside in the same household as you and had been diagnosed with COVID-19 (via nucleic acid or antigen testing) at any time prior to the survey?
2. Yes

B. No

1. Have you purchased COVID-19 drugs?
2. Yes
3. B. No (Skip to Question 12)
4. Which of the following COVID-19 drugs have you purchased? (Multiple choices allowed)
5. Medicines for relieving fever and pain, such as ibuprofen, Lianhua Qingwen, aspirin, acetaminophen-containing drugs (e.g., Sanlitong, Paracetamol)
6. Medicines for relieving cold symptoms like runny nose, stuffy nose, and sneezing, such as Tylenol, Baijiahei, Vitamin C Yinqiao Pian
7. Medicines for relieving cough and phlegm, such as Chuanbei Pipa Gao, Pipa Lu, Jizhi Tangjiang, Glycyrrhiza Oral Liquid, Feilike
8. Medicines for relieving digestive symptoms like nausea, vomiting, and diarrhea, such as Huoxiang Zhengqi Ye/Capsules, antidiarrheals

E. Other medicines

11.Through what channel did you successfully purchase these medicines? (Multiple choices allowed)

1. Hospitals
2. Pharmacies
3. Online platforms

D. Other channels

12.Why haven't you purchased Covid-19 medicines to respond to COVID-19?

1. I have tried, but were unable to access any COVID-19 medicine

B. I don’t think it is necessary to buy any COVID-19 medicine

1. In the past week, has the adjustment of COVID-19 policies affected your sleep?
2. Yes
3. No (Skip to Question 16)
4. How do you rate the level of the impact of zero-COVID policy adjustment on your sleep status?
5. Very significant
6. Significant
7. Moderate
8. Slight

15.What do you think are the specific reasons for your sleep disturbance under the scenario of zero-COVID policy adjustment? (Multiple choices allowed)

1. Worry about insufficient supplies (e.g., medicines) for COVID-19 response
2. Worry about getting infected
3. Worry about family members getting infected
4. Worry about the pandemic affecting work,business, study, et al
5. Other: ______

16.How likely do you think you or your family members are to be infected with COVID-19 in the next two months?

1. Definitely will be infected
2. Highly likely to be infected
3. May be infected
4. Unlikely to be infected
5. Definitely will not be infected

17.If you test positive at home (via nucleic acid or antigen testing) for COVID-19, what will you do?

1. Go to the hospital immediately
2. Stay at home, and go to the hospital until symptoms appear

C. Stay at home, and go to the hospital only if symptoms become serious and can’t be improved by self-medication.

1. In the past two weeks, have you engaged in any of the following behaviors? (Multiple choices allowed)
2. Frequently checked COVID-19-related information
3. Washed hands frequently
4. Became more strict about wearing masks
5. Tried every means to buy more related items (e.g., masks, antigen test kits, medicines)
6. Changed travel methods, reducing the use of public transportation
7. Received COVID-19 vaccination
8. Tried to stay at home and not go out
9. Conducted repeated checks (e.g., nucleic acid testing, temperature measurement, medical visits)

I. None of the above
